# Enhancing breast milk production with Domperidone in mothers of preterm neonates (EMPOWER trial)

**DOI:** 10.1186/1471-2393-12-87

**Published:** 2012-08-31

**Authors:** Elizabeth V Asztalos, Marsha Campbell-Yeo, Orlando P daSilva, Alex Kiss, David C Knoppert, Shinya Ito

**Affiliations:** 1 Department of Newborn & Developmental Paediatrics, University of Toronto, Centre for Mother, Infant, Child Research, Sunnybrook Research Institute, Sunnybrook Health Sciences Centre, 2075 Bayview Avenue, Toronto, Ontario, M4N 3M5, Canada; 2 IWK Health Centre, Dalhousie University, Halifax, NS, Canada; 3 Perinatal and Women’s Health, London Health Sciences Centre, London, ON, Canada; 4 Sunnybrook Research Institute, University of Toronto, Toronto, Canada; 5 Pharmacy Services, London Health Sciences Centre, London, Canada; 6 Department of Pharmacology, Hospital for Sick Children, University of Toronto, Toronto, ON, Canada

**Keywords:** Domperidone, Galactogogues, Mothers of preterm infants, Breast milk

## Abstract

**Background:**

The use of ***mother’s own breast milk*** during initial hospitalization has a positive impact not only in reducing potential serious neonatal morbidities but also contribute to improvements in neurodevelopmental outcomes. Mothers of very preterm infants struggle to maintain a supply of breast milk during their infants’ prolonged hospitalization. Galactogogues are medications that induce lactation by exerting its effects through oxytocin or prolactin enhancement. Domperidone is a potent dopamine D_2_ receptor antagonist which stimulates the release of prolactin. Small trials have established its ability in enhancing breast milk production. EMPOWER was designed to determine the safety and efficacy of domperidone in mothers experiencing an inadequate milk supply.

**Methods/design:**

EMPOWER is a multicenter, double masked, randomized controlled phase-II trial to evaluate the safety and effectiveness of domperidone in those mothers identified as having difficulty in breast milk production. Eligible mothers will be randomized to one of two allocated groups: **Group A**: domperidone 10 mg orally three times daily for 28 days; and **Group B**: identical placebo 10 mg orally three times daily for 14 days followed by domperidone 10 mg orally three times daily for 14 days. The primary outcome will be determined at the completion of the **first 2-week period**; the second 2-week period will facilitate answering the secondary questions regarding timing and duration of treatment. To detect an estimated 30% change between the two groups (from 40% to 28%, corresponding to an odds ratio of 0.6), a total sample size of 488 mothers would be required at 80% power and alpha = 0.05. To account for a 15% dropout, this number is increased to 560 (280 per group). The duration of the trial is expected to be 36–40 months.

**Discussion:**

The use of a galactogogue often becomes the measure of choice for mothers in the presence of insufficient breast milk production, particularly when the other techniques are unsuccessful. EMPOWER is designed to provide valuable information in guiding the practices for this high-risk group of infants and mothers. The results of this trial will also inform both mothers and clinicians about the choices available to increase and maintain sufficient breast milk.

**Trial registration:**

Clinical Trials.gov Identifier: NCT01512225

## Background

Despite the recent perinatal and neonatal technological advances, at least 8.2% of births in Canada are preterm (<37 weeks gestation), a figure that has continued to rise [1,2]. The very preterm infant is often growth restricted because of the concurrent illnesses and difficulties in optimizing energy and nutrient intake for normal growth [3]. Studies have suggested that early nutritional intervention (breast milk and preterm formula) that will support growth, in particular brain growth, will lead to an improved outcome even in the presence of injury [4-6]. There is further evidence to suggest that the use of ***mother’s own breast milk*** compared to infant formula during initial hospitalization has a positive impact not only in reducing potential serious neonatal morbidities but also contribute to improvements in neurodevelopmental outcomes [6-12]. There is a growing movement in neonatal care for very preterm infants to receive breast milk as the primary source of nutrition rather than rely on preterm formula. Mothers are therefore encouraged to begin pumping their breasts to provide breast milk for their infants. With preterm infants requiring hospitalization for anywhere from 10–16 weeks before their discharge from initial hospitalization, continued and sustained breast milk volume can prove to be a challenge to even the most dedicated of mothers. Most mothers of very preterm infants, for a variety of reasons such as illness, stress and other factors related to preterm birth, are unable to express sufficient amounts of milk to exclusively feed their infants [11-15].

There have been many identified and studied non-pharmacological measures such as emotional support, kangaroo care, skin-to-skin contact, expressing breast milk at the infant’s bedside, increasing pumping frequency and duration and types of mechanical expression that have been found to contribute to variable levels of success in augmenting the breast milk production in mothers of preterm infants [16]. For those mothers in whom milk production has declined and is not responding to non-pharmacologic measures, the use of galactogogues is often considered. Galactogogues are medications that induce lactation generally from exerting its effects through oxytocin or prolactin enhancement [17]. The primary galactogogues used today for prolactin enhancement are dopamine antagonists with the most widely studied being metoclopramide and domperidone. Domperidone is a potent dopamine D_2_ receptor antagonist and was developed and marketed as a prokinetic and antiemetic agent. By blocking dopamine D_2_ receptors in the anterior pituitary, domperidone stimulates the release of prolactin Table 1[18-23].

**Table 1:** Outlines the series of small trials that have evaluated the efficacy of domperidone in mothers of preterm infants

**Study Domperidone**	**N**	**Placebo**	**Randomization**	**Intervention**	**Findings**
De Leo [18]	15	Y	N	10 mg TID 4d	↑ lactation
Petraglia [19]	17	Y	N	10 mg TID 10d	↑ PRL, BM
da Silva [20]	20	Y	Y	10 mg TID 7d	↑ PRL, BM
Campbell-Yeo [21]	46	Y	Y	10 mg TID 14d	↑↑ PRL, BM
Ingram [22]	80	Metoclopromide	Y	10 mg TID 10d	↑ BM
Knoppert [23]	15	N	Y	10 mg vs. 20 mg TID 4 weeks	↑ BM

### Rationale for phase II trial

Mothers of preterm infants are at a higher risk of a decline in milk production. It has been recommended that they should be evaluated no later than two weeks postpartum to determine what intervention may be required to assist them in augmenting and maintaining an adequate supply of milk [24]. Although other measures can and should be considered, the use of a galactogogue often becomes the measure of choice, particularly when the other techniques are unsuccessful. The limited body of research on domperidone suggests that it has potential as a galactogogue in mothers of preterm infants; this has led to widespread use of this medication. However, there is a paucity of research to best guide clinicians as to the optimal dose, timing of administration and duration of treatment and to reaffirm safety. EMPOWER was initiated to firmly establish the safety of domperidone for mothers of preterm infants but also guide clinicians regarding initiation, timing and duration of treatment.

## Methods/design

### Primary research question

The primary research question is: in mothers of preterm infants 23–29 completed weeks (23 1/7-29 6/7 weeks) gestation at birth who are pumping to provide expressed breast milk for their infant(s) and are identified as having an inadequate milk supply, does the administration of domperidone compare to placebo increase breast milk volume without any signs of harm over a 2 week period?

### Secondary research questions

There are several secondary research questions:

i. Is there a relationship between the volume of breast milk produced and timing of initiation and duration of domperidone to treat mothers with defined low breast milk supply?

ii. Does gestation at birth contribute to any treatment effect from domperidone in mothers with defined low breast milk supply?

iii. Is there a difference in the volume of breast milk produced in mothers identified having an inadequate supply within 7–14 days post delivery versus mothers identified 15–21 days post delivery?

iv. Is there a difference in the use of supplementation such as donor breast milk or preterm infant formula between the different strategies of administration during the study period?

v. Is there a difference in the use of supplementation, such as formula or donor breast milk by the mother to her infant at 40 weeks post conceptual age (term) and at 6 weeks corrected age (6 weeks post term) between the two groups?

vi. Is there a difference in potential adverse events or effects, in particular, those related to gastrointestinal or cardiac difficulties between the two approaches?

### Design

**Figure 1:**
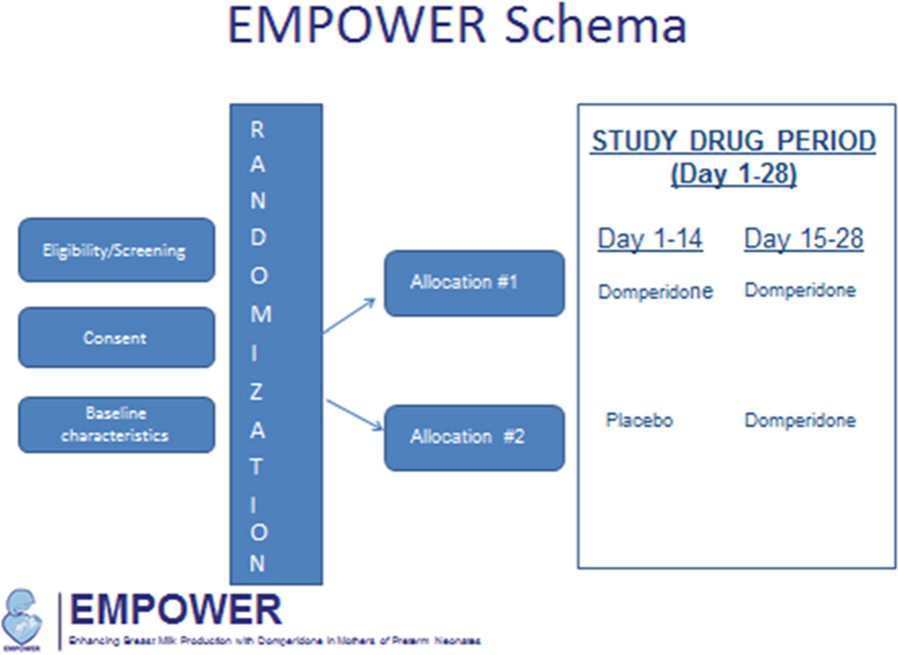
EMPOWER Schema.

EMPOWER is a multi-centre, double-masked, randomized controlled trial in which 560 mothers will randomized to one of two allocated groups: **Group A**: domperidone 10 mg orally three times daily for 28 days; and **Group B**: identical placebo 10 mg orally three times daily for 14 days followed by domperidone 10 mg orally three times daily for 14 days. (Figure 1) The primary outcome will be determined at the completion of the **first 2-week period** comparing study drug and placebo; the second 2-week period will facilitate answering the secondary questions regarding timing and duration of treatment. The mothers will be contacted when their infants are term gestation (40 weeks post conceptual age) and 6 weeks corrected (6 weeks post date of confinement) to document the feeding patterns (breastfeeding/bottle feeding), continued frequency of breast stimulation, additional use of a galactogogue or alternative after the study period, and the use of supplementation other than mother’s own milk for their infants at these time points. In addition, the mothers will be asked to a few questions regarding the intervention and their participation in the trial.

### Randomization

Upon consent, the site co-ordinator will be able to randomize the mother using a 24 hour/day web-based randomization service at the data coordinating centre (DCC) at the Centre for Mother, Infant, and Child Research (CMICR) in Toronto. A treatment number will be issued which will correspond to the study medication at the centre which has been previously sent to the site. The study allocation will be randomly assigned in a 1:1 ratio in blocks of 4 and 8. The study participants will be stratified by centre, gestational age groupings (23–26 and 27–29 weeks gestation at delivery) and days post delivery (7–14 and 15–21 days post delivery).

### Study setting

The study setting is multi-national with approximately 20–25 centres in Canada, Israel, Qatar, and Chile. Enrollment began in June 2012 and is expected to be completed in 36–42 months.

### Ethics, informed consent and safety

Documented approval has been obtained from the Research Ethics Boards/Institutional Review Boards of the participating centres prior to study start. In addition documented approval has been obtained from Health Canada to study domperidone for this off-label indication. The study is also designed to conform to the International Conference on Harmonization of good clinical practice (GCP) guidelines [25], as well as local regulations and policies as well as with the Declaration of Helsinki. Written informed consent must be obtained from each participant.

There is no interim analysis planned but regular safety reviews are scheduled for every 100 patients recruited at which time all adverse and serious adverse events are reviewed by the Data Safety Monitoring Board (DSMB). The DSMB will also have the right to review any variables that may have an impact on the trial.

### Eligibility

#### Inclusion criteria

The goal is to identify those mothers truly at higher risk of not being able to produce and maintain a supply of breast milk in sufficient quantities for her infant.

i. mothers of a preterm infant born ≤ 29 completed weeks gestation (23 1/7-29 6/7 weeks)

ii. postpartum period of 7–21 days

iii. mechanically pumping an minimum average of 6 times a day in the 4–7 days prior to entry

iv. experiencing inadequate milk supply defined as providing <100% of the average of the daily nutritional intake during the previous 72 hour period prior to entry based on a fluid intake of 150 ml/kg/d or experiencing a clinical reduction of 30% from a peak volume during the previous 72 hour period prior to entry (maternal report). The 30% reduction reported by the mother will be confirmed by lactation support personnel in the NICU. In the case of mothers with twins, the estimated fluid volume to be produced will be based on the weight of the larger twin. Mothers can be identified in the NICU as early as day 4 until day 18 post delivery.

### Exclusion criteria

i. history of known or suspected cardiac dysrhythmias (tachyarrhythmia, Q-Tc prolongation) or currently on an anti-arrhythmic medication

ii. currently experiencing mastitis

iii. previous breast surgery, including augmentation or reduction, nipple piercing

iv. known chronic or debilitating illness, known abnormal liver function, gastric abnormalities (gastrointestinal hemorrhage, blockage or currently treated acid reflux), HIV

v. known to a have prolactin-releasing pituitary tumor

vi. receiving medications known to alter the metabolism and pharmacokinetics of domperidone (eg. oral “azole” antifungals, erythromycin antibiotics, MAO inhibitors) or medications that have dopaminergic or antidopaminergic activity or affect prolactin levels

vii. mother of higher order pregnancies (triplet, or more)

viii. current cigarette smoking (cigarette smoking is known to diminish prolactin levels)

### Duration of study period

The total study period for the study medication is 4 weeks (28 days). The study medication will end prior to 28 days if any one of the following occurs:

i.  if the infant dies during the study period

ii.  mother’s refusal for ongoing participation with the protocol

iii.  if the mother is experiencing any irregular heartbeats (documented by an ECG)

iv.  if the Q-Tc interval in the ECG is > 0.44 sec (based on standard ECG run at 25 mm/sec) for the mother

v.  if the infant is demonstrating an irregular heartbeat or prolonged Q-Tc interval confirmed by ECG; blood for domperidone level will be drawn on the infant only if this occurs.

### Study outcomes

#### Primary outcome

The primary outcome is the difference between the two groups in achieving a 50% increase in breast milk volume at the end of the first 2-week period (mean day 14 volume- mean day 0 volume at entry).

#### Secondary outcomes

The secondary outcomes include:

i. mean difference in the tabulated volume in breast milk recorded between the two groups at 2 and 4 weeks (mean day 14 or 28 volume- mean day 0 volume at entry)

ii. mean within-subject differences between the two groups at 2 and 4 weeks (mean day 14 or 28 volume- mean day 0 volume at entry)

iii. effect of gestational age at the time of delivery on the volume of milk (23–26 and 27–29 weeks gestation at delivery) between the two groups

iv. effect of timing of inadequate milk supply post delivery on the volume of milk (7–14 and 15–21 days) between the two groups

v. difference in the use of supplementation to expressed breast milk, such as formula or donor breast milk between the two groups during the study period (day 14 and 28)

vi. rates of breastfeeding, use of supplementation, such as formula or donor breast milk at 40 weeks post conceptual age (term) and at 6 weeks post term between the different groups

vii. potential differences in adverse events, in particular, those related to gastrointestinal or cardiac difficulties

### Statistical analysis

All analyses will be carried out using SAS Version 9.1 (SAS Institute, Cary, NC, USA). Descriptive statistics will be calculated for all variables of interest. Continuous measures will be summarized using means and standard deviations whereas categorical measures will be summarized using counts and percentages.

The primary outcome will be assessed between groups using a logistic regression model. This model will account for correlation among observations taken at the same centre as well as multiple births by the same mother using generalized estimating equations. The model will take into account variables such as gestational age and days post delivery. The number of variables included in the model will be determined using regression modeling guidelines (i.e. for logistic models, the maximum number of variables allowed will equal the number of observations in the smallest of the two outcome categories divided by 10). Prior to analysis, variables will be assessed for the presence of multi-collinearity (tolerance statistic < 0.4), and in this case, only one member of a correlated set will be retained in the model.

The secondary outcomes involving mean difference in the tabulated volume in breast milk will be assessed using a repeated measure analysis of covariance comparing the different groups across the time points of interest, adjusting for correlation among observations taken at the same centre and on the same subject. The model will assess differences between groups, differences over time (within-subject differences), and the group by time interaction adjusting for gestational age and days post delivery.

A linear regression model will be run to assess the relationship between gestational ages at time of delivery on the volume of milk. The model will include group, gestational age (23–26 weeks, or 27–29 weeks), as well as the group by age interaction term. Another linear regression model will be run to assess the relationship between timing of inadequate milk supply post delivery on the volume of milk. The model will include group, timing (7–14 days, 15–21 days), as well as the group by timing interaction term. All regression models conducted in this study will be run using likelihood based methods to account for missing data. The models will also treat twins or triplets as a correlated set of data to adjust for their dependent nature.

Use of supplementation, rates of breastfeeding, and adverse events will be assessed using a chi-square analysis comparing groups.

## Discussion

Currently, there is no clear approach in managing mothers of preterm infants experiencing an inadequate production of breast milk. With EMPOWER, we hypothesize that domperidone, through its pharmacologic action on increasing prolactin levels, will assist mothers of very preterm infants, experiencing inadequate breast milk production, in increasing breast milk volumes to a level identified as being sufficient for continued pumping in the hospitalization period. We also hope to determine the safety and efficacy of domperidone in helping a mother who is experiencing an inadequate milk supply, and how it should be considered in the care of the mother and her preterm infant without causing adverse consequences to either the mother or infant.

## Competing interests

All of the authors listed have nothing to disclose.

## Authors’ Contributions

EVA, MCY, ODS are the principal investigators on the EMPOWER trial and were extensively involved in the study concept and design. AK is the biostatistician for the trial and involved in the development of the analysis plan. DK and SI provide the clinical support from a pharmacological perspective. All of the authors have extensively reviewed the protocol.

## Funding

EMPOWER is funded by a 5-year operating grant from the Canadian Institutes of Health Research.

## Pre-publication history

The pre-publication history for this paper can be accessed here:

http://www.biomedcentral.com/1471-2393/12/87/prepub
